# LILRB4 Decrease on uDCs Exacerbate Abnormal Pregnancy Outcomes Following *Toxoplasma gondii* Infection

**DOI:** 10.3389/fmicb.2018.00588

**Published:** 2018-03-28

**Authors:** Shaowei Zhan, Jing Zheng, Haixia Zhang, Mingdong Zhao, Xianbing Liu, Yuzhu Jiang, Chunyan Yang, Liqin Ren, Zhiqiang Liu, Xuemei Hu

**Affiliations:** ^1^Department of Gynecology and Obstetrics, Binzhou Affiliated Hospital of Binzhou Medical University, Binzhou, China; ^2^Department of Gynecology and Obstetrics, Yantai Traditional Chinese Medicine Hospital, Yantai, China; ^3^Department of Immunology, Department of Medicine & Pharmacy Research Center, Binzhou Medical University, Yantai, China; ^4^Department of Radiology, Binzhou Affiliated Hospital of Binzhou Medical University, Binzhou, China

**Keywords:** LILRB4, dendritic cells, co-stimulatory molecules, abnormal pregnancy outcomes, *Toxoplasma gondii*

## Abstract

*Toxoplasma gondii* (*T. gondii*) infection in early pregnancy can result in miscarriage, dead fetus, and other abnormalities. The LILRB4 is a central inhibitory receptor in uterine dendritic cells (uDCs) that plays essential immune-regulatory roles at the maternal–fetal interface. In this study, *T. gondii*-infected human primary uDCs and *T. gondii*-infected LILRB4^-/-^ pregnant mice were utilized. The immune mechanisms underlying the role of LILRB4 on uDCs were explored in the development of abnormal pregnancy outcomes following *T. gondii* infection *in vitro* and *in vivo*. Our results showed that the expression levels of LILRB4 on uDCs from normal pregnant mice were obviously higher than non-pregnant mice, and peaked in mid-gestation. The LILRB4 expression on uDC subsets, especially tolerogenic subsets, from mid-gestation was obviously down-regulated after *T. gondii* infection and LILRB4 decrease could further regulate the expression of functional molecules (CD80, CD86, and HLA-DR or MHC II) on uDCs, contributing to abnormal pregnancy outcomes. Our results will shed light on the molecular immune mechanisms of uDCs in abnormal pregnancy outcomes by *T. gondii* infection.

## Introduction

*Toxoplasma gondii* (*T. gondii*) is an obligate intracellular parasite capable of infecting a wide range of mammalian hosts including humans. Infection by *T. gondii* during pregnancy can cause severe sequelae, such as spontaneous abortion, stillbirth, low birth weight, and significant birth defects for surviving neonates (Robbins et al., 2012). Although several immune mechanisms have been postulated (Xu et al., 2013; Zhao et al., 2013; Liu Y. et al., 2014), the detailed mechanisms underlying adverse pregnancy outcomes following *T. gondii* infections need to be further explored. The microenvironment at the maternal–fetal interface plays an important role in maintaining normal pregnancy (Hunt and Robertson, 1996). Multiple immune cells and cytokines at the maternal–fetal interface participate in protecting the semi-allogeneic embryo from maternal attack and promote immune tolerance during pregnancy (Guleria and Sayegh, 2007). Among these immunocompetent cells at the maternal–fetal interface, antigen-presenting cells (APCs) are regarded as important participants in immune regulation during pregnancy (Della Bella et al., 2011).

Dendritic cells are essential for the initiation of primary immune responses and have been reported to induce immunological tolerance and to regulate cell-mediated immune responses (Langenkamp et al., 2000). Uterine dendritic cells (uDCs) are scattered throughout the decidualized endometrium throughout gestation and play vital immune-regulatory role at the maternal fetal interface (Laskarin et al., 2007). In normal human early pregnancy, uDCs include BDCA-1^+^CD19^-^CD14^-^ myeloid DC type 1 (MDC1), BDCA-3^+^CD14^-^ myeloid DC type 2 (MDC2), and BDCA-2^+^CD123^+^ plasmacytoid DC (PDC) subsets (Ban et al., 2008). MDCs have been reported to induce certain forms of immunity responsible for the maintenance of a normal pregnancy (Gardner and Moffett, 2003). Mice uDCs have been classified into two distinct subsets, CD11c^+^CD8α^-^ and CD11c^+^CD8α^+^. The CD11c^+^CD8α^-^ subset belongs to the myeloid lineage and comprises the vast majority of uDCs, mainly exhibiting an immature phenotype and contributing to the induction of maternal–fetal immune tolerance (Blois et al., 2004).

In the periphery, tolerogenic status for DCs is characterized by a low level of the co-stimulatory molecules CD80 and CD86 and high expression of the inhibitory receptors LILRB4 (also called ILT3, gp49B, CD85k) (Adorini et al., 2004). The inhibitory receptor LILRB4, which is mainly expressed on professional APCs, belongs to immunoglobulin superfamily members and contains an immune-receptor tyrosine-based inhibitory motif (ITIM) in their intracellular domains to transduct negative signals (Cella et al., 1997; Kim-Schulze et al., 2006). LILRB4-expressing APC plays prominent roles in controlling inflammation by inhibiting the expression of co-stimulatory molecules (Chang et al., 2009; Vlad et al., 2010). Further evidence indicated that over-expression of LILRB4 can inhibit the transcription of NF-κB-dependent genes that encode co-stimulatory molecules (CD80, CD86) in DCs (Cella et al., 1997). Functional studies have suggested that LILRB4 neutralization can enhance antigen presentation (Regnault et al., 1999; Kasai et al., 2008). At the fetal–maternal interface, *in vivo* studies showed that LILRB4 mRNA has been detected in murine uterine endometrium during early pregnancy (Matsumoto et al., 1997), and LILRB4 protein expression was detected on immature uDCs of human decidual tissue using flow cytomety (Ban et al., 2008).

Our previous study showed that uDCs contribute to abnormal pregnancy outcomes caused by *T. gondii* infection in early pregnancy (Liu X. et al., 2014). Most importantly, our recent study has reported that LILRB4 on decidual macrophage was involved in the development of abnormal pregnancy outcomes during *T. gondii* infection (Li et al., 2017). Whether LILRB4 on uDCs also contributed to abnormal pregnancy outcomes after *T. gondii* infection remains unclear, and the associated mechanisms are also unknown. Hence, in the present study, *T. gondii-*infected human uDCs and *T. gondii-*infected LILRB4^-/-^ pregnant mice were used to explore the mechanisms related to LILRB4 on uDCs that lead to abnormal pregnancy outcomes.

## Materials and Methods

### Maintenance of *T. gondii* Tachyzoites (RH Strain)

The *T. gondii* tachyzoites were cultured in HEp-2 cells in Minimum Essential Media (MEM) (Hyclone, United States), 5% fetal bovine serum (FBS; Gibco, United States), and 100 IU/ml penicillin/streptomycin (Sigma-Aldrich, United States). After culture, tachyzoites were centrifuged at 1500 rpm (433 ×*g*) for 10 min, and purified tachyzoites were resuspended in MEM and counted using a Neubauer chamber. The experiment was carried out in BSL-2 laboratories. All the liquids, consumables and labwares contaminated with the parasites were collected and steeped immediately in disinfectant, and sterilized by high-pressure sterilizer. The mice carcasses were collected in ice locker and transported out by the professionals of public health agencies.

### Human Abortion Sample Collection

Abortion samples of the first-trimester decidua (6–12-week gestation) from elective termination procedures were obtained from Yantai Affiliated Hospital of the Binzhou Medical University. All patients signed an informed consent form before enrollment, and sample collection for this study was approved by the ethics Committee of Binzhou Medical University. The decidual tissues were rinsed in sterile saline solution and rapidly transferred to ice-cold Roswell Park Memorial Institute medium (RPMI). All samples were disposed as soon as possible. Human sample collection procedures for this study were approved by the Binzhou Medical University Ethics Committee, and all patients provided written informed consent for the collection of samples and subsequent analysis.

### Human Decidual Cell Preparation and Flow Cytometry Analysis

Decidual tissues were washed 4–5 times with RPMI medium. Pieces of decidual tissue were minced using the gentle MACS^TM^ Dissociator (Mitenyi-Biotec, Germany) according to the manufacturer’s instructions, and then digested in 0.1% collagenase type IV and 25 IU/ml DNase I (both from Sigma-Aldrich, St. Louis, MO, United States) in RPMI for 45 min at 37°C with gentle rotation. Single cell suspension was filtered twice with a 75 μm pore size nylon mesh (Miltenyi Biotec GmbH, Bergisch Gladbach, Germany) and centrifuged at 2000 rpm (771 ×*g*) for 10 min at room temperature. The mononuclear cells were then isolated by density gradient separation over a standard Ficoll-Hypaque Lymphoprep (1.077, Haoyang Biological Manufacture, Co., Tianjin, China). Decidual mononuclear cells were collected from the interface and were washed twice in cold phosphate buffer solution (PBS). Then the cells were incubated for 12 h in RPMI supplemented with 10% FBS, 100 IU/ml penicillin, and 100 IU/ml streptomycin in a humidified incubator at 37°C with 5% CO_2_. After 12 h of culture, the cells for the infected group were co-cultured with *T. gondii* tachyzoites at a ratio of 1:2 for 12 h in 6-well culture plates. The LILRB4-neutralized infected group was infected at the same condition in the presence of anti-IILRB4 neutralizing antibodies (mAbs) (10 μg/mL, eBioscience, United States). The uninfected group was considered as control. Cells were incubated for 12 h in the same condition as described above. The mononuclear cells were collected and stained with fluorophore-conjugated mAbs: FITC-conjugated anti-CD1c (BDCA-1), FITC-conjugated anti-CD303 (BDCA-2), FITC-conjugated anti-CD141 (BDCA-3) (all from Miltenyi Biotec GmbH, Bergisch Gladbach, Germany), PerCP-Cy5.5-conjugated anti-CD14, CD19, CD123, PE-conjugated anti-HLA-DR, PE-conjugated anti-CD80, PE-conjugated anti-CD86 (all from BD Pharmingen, United States), and APC-conjugated anti-LILRB4 (eBioscience, United States). The mAbs were added according to the manufacturer’s protocol. After incubation, the cells were washed twice with PBS and resuspended in 600 μl of PBS. The cells were detected using BD FACSAria flow cytometry (Becton Dickinson, United States) and the data were analyzed using BD FACSDiva 7.0 software (Becton Dickinson).

### Animal Models

Wild type (WT) female mice (Beijing Weitong Lihua Experimental Animal Technical, Co., Ltd.) and LILRB4^-/-^ female mice (Experimental Animal Division RIKEN BioResource Center, Japan) at 6- to 8-week-old were mated with the corresponding 8- to 10-week-old male mice overnight at a ratio of 2:1 and were checked for vaginal plugs the next morning. Females with a vaginal plug were segregated and designated as gestational day 0 (Gd 0). The infected group were inoculated intraperitoneally (i.p.) with 400 tachyzoites in 200 μl sterile PBS on Gd 8. The uninfected groups were inoculated with equivalent PBS at the same time. The protocol of animal experiment was approved by the Committee on the Ethics of Animal Experiments of the Binzhou Medical University. All procedures were performed under sodium pentobarbital anesthesia, and all efforts were made to minimize suffering of the animals.

### Mice Mononuclear Cell Preparation and Flow Cytometry

Mononuclear cells from mouse uterine and placenta were prepared as previously described (Liu X. et al., 2014). Briefly, mice were sacrificed on Gd 14. The uterus and placenta were excised with surgical cuts. The pregnancy outcome was reflected by the total number of fetuses, fetal size, stillbirth, absorptive fetus, and hemorrhagic appearance. The uterus and placentas were washed with sterile PBS, minced into small pieces. Dispersed cells were collected by filtration through a 48 μm pore size stainless steel mesh. Thereafter, the mononuclear cells were obtained by density-gradient centrifugation and washed twice in cold PBS. The following fluorochrome-conjugated, mouse-specific mAbs were used in the assays: FITC-conjugated anti-CD11c (BD Biosciences, United States), Percp-cy5.5-conjugated anti-CD8a mAb (BD Biosciences, United States), PE-conjugated anti-CD80 (BD Biosciences, United States), PE-conjugated anti-CD86 (BD Biosciences, United States), PE-conjugated anti-I-Ad/I-Ed (MHC II) (BD Biosciences, United States), and PE-conjugated anti-LILRB4 (Biolegend, United States). Cells were incubated for 30 min at 4°C in the dark according to the manufacturer’s instructions. Subsequently, the cells were washed with cold PBS and analyzed using BD FACSAria flow cytometry and BD FACSDiva 7.0 software (Becton Dickinson, United States).

### Statistical Analysis

Data were presented as the mean ± SEM. Statistical analysis was performed using the SPSS statistics software package (SPSS 17.0; SPSS, Inc., Chicago, IL, United States). Unpaired *t*-tests were used after verifying that the data of the groups had a normal distribution by SAS to compare two independent groups. *p* < 0.05 was regarded as significant and *p* < 0.01 was considered as very significant.

## Results

### The Abnormal Pregnancy Outcomes Were Related to LILRB4 in *T. gondii* Infected Mice

**FIGURE 1:**
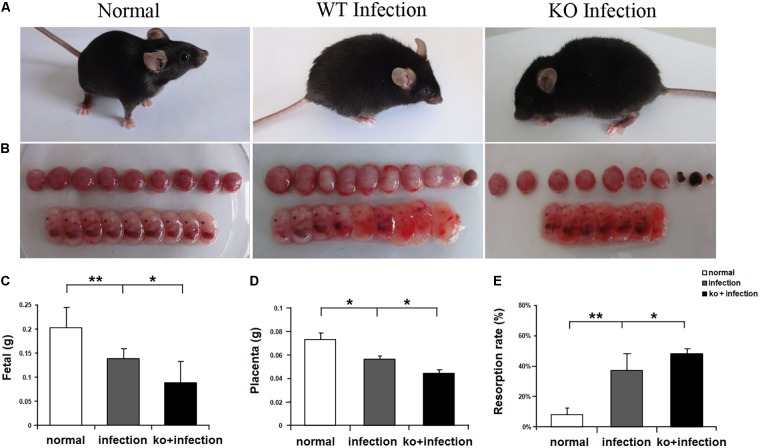
The impact of LILRB4 on abnormal pregnancy outcomes caused by *Toxoplasma gondii* infection. **(A)** The pregnant mice and **(B)** their fetuses from normal wild type (WT) pregnant, *T. gondii* infected WT pregnant mice, and *T. gondii* infected LILRB4^-/-^ pregnant mice. **(C)** The weight of fetuses, **(D)** the weight of placentas, and **(E)** the resorption rate from the three groups. Data are shown as means ± SD for 10 pregnant mice and differences were identified using unpaired *t*-tests (^∗^*p* < 0.05, ^∗∗^*p* < 0.01).

*Toxoplasma gondii* infection during early pregnancy can cause abnormal pregnancy outcomes. In order to explore the effects of LILRB4 on the adverse pregnancy outcome caused by *T. gondii*, we observed the pregnancy outcomes of *T. gondii* infected LILRB4^-/-^ mice. The results were in accordance with our previous studies (Li et al., 2017). In both the WT and LILRB4^-/-^ infected pregnant group, the pregnant mice were flagging, shambling, and had erected fur, whereas the control mice were nimble, restive, and had smooth pelage (**Figure 1A**). Compared with WT infection groups, most fetuses from LILRB4^-/-^ infected pregnant mice were evidently smaller and shapeless, placentas were obvious more hemorrhagic and resorbed (**Figure 1B**). The weight of fetus or placenta from LILRB4^-/-^ infection group was less than that of WT infection group respectively (**Figures 1C,D**). The abortion rate of LILRB4^-/-^ infected group was significantly increased than that of WT infection group (**Figure 1E**). Thus, the knockout of LILRB4 aggravate the abnormal pregnancy outcome caused by *T. gondii* infection.

### The Dynamic LILRB4 Expression on Murine uDCs in Normal Pregnancy

**FIGURE 2:**
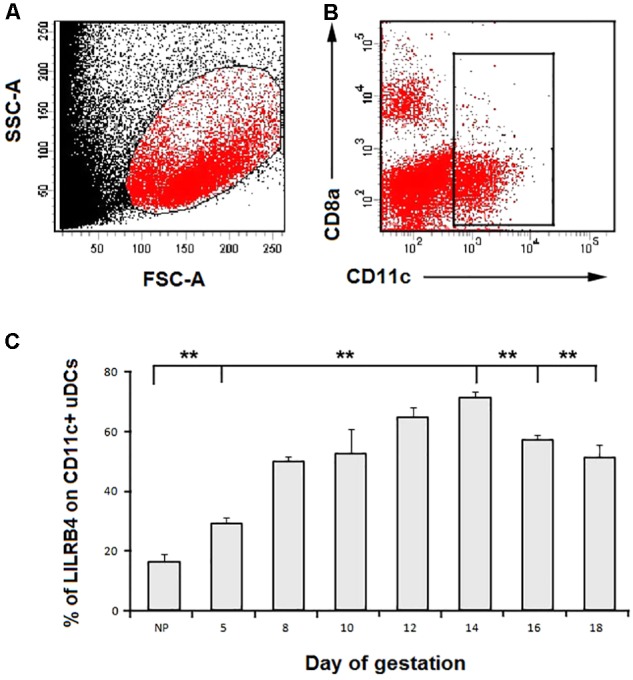
Dynamic expression of LILRB4 on uterine DCs. **(A)** Decidual lymphocytes were gated in the appropriate location for flow cytometric analyses. **(B)** Uterine DCs were gated by CD11c expression. **(C)** Flow cytometry analyses of LILRB4 dynamic expression on CD11c^+^ uterine DCs were performed using non-pregnant mice and normal pregnant mice at day of gestation (Gd) 5, 8, 10, 12, 14, 16, 18, respectively. Data are shown as means ± SD for 10 pregnant mice and differences were identified using unpaired *t*-tests (^∗^*p* < 0.05, ^∗∗^*p* < 0.01).

CD11c^+^ cells were gated as uDCs shown in **Figures 2A,B**. LILRB4 expression on uDCs of normal pregnant mice at Gd 5, 8, 10, 12, 14, 16, 18, and non-pregnant mice was detected using flow cytometry (**Figure 2C**). The results showed that LILRB4 expression was at a lower level on uDCs from non-pregnant mice compared with the normal pregnant mice at all the designed time points. During pregnancy, LILRB4 expression gradually increased from Gd 5 to Gd 14, peaked on Gd 14, and decreased after Gd 14.

### LILRB4 Expression on uDCs Subsets in Both Mice and Human Was Down-Regulated After *T. gondii* Infection

**FIGURE 3:**
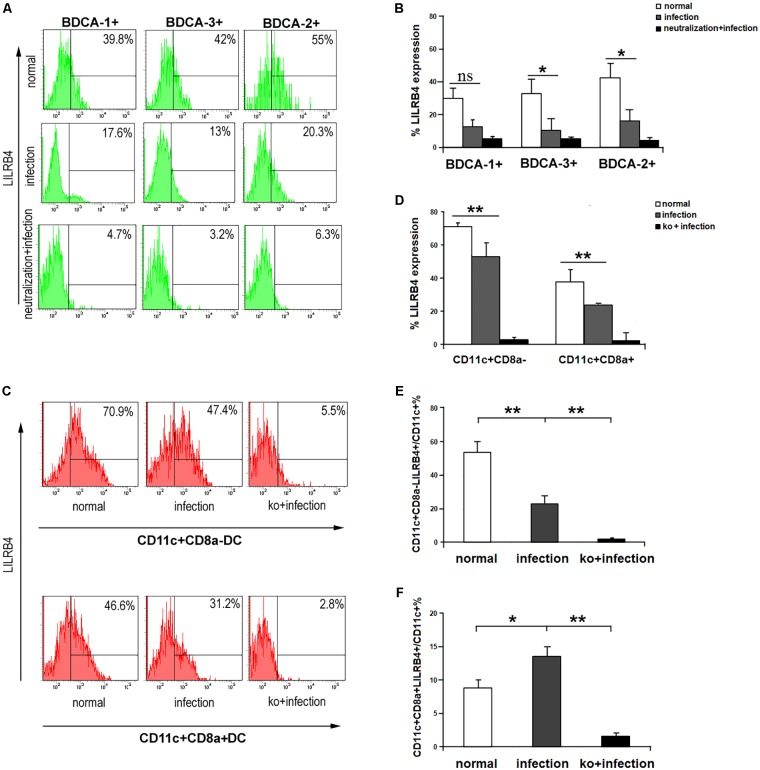
Expression of LILRB4 on uDC subsets was down-regulated following *T*. *gondii* infection. **(A)** Flow cytometry analyses of LILRB4 expression on BDCA-1^+^(BDCA-1), BDCA-2^+^(BDCA-2), and BDCA-3^+^(PDC) uDC subsets were performed in normal, infected, and LILRB4-neutralized infected human uDCs. **(B)** LILRB4 expression changes were compared after *T*. *gondii* infection in human BDCA-1^+^, BDCA-2^+^, and BDCA-3^+^ uDC subsets from normal, infected, and LILRB4-neutralized infected groups. **(C)** Flow cytometry analyses of LILRB4 expression in CD11c^+^CD8a^+^ and CD11c^+^CD8a^-^ uDC subsets were performed in normal, infected, and LILRB4^-/-^ infected mice. The changes of LILRB4 expression compared between CD11c^+^CD8a^+^ and CD11c^+^CD8a^-^ uDC subsets were showed as **(D)** CD11c^+^CD8a^-^LILRB4^+^/CD11c^+^CD8a^-^% or CD11c^+^CD8a^+^LILRB4^+^/CD11c^+^CD8a^+^%, **(E)** CD11c^+^CD8a^-^LILRB4^+^/CD11c^+^%, **(F)** CD11c^+^CD8a^+^LILRB4^+^/CD11c^+^%. Data are shown as means ± SD (^∗^*p* < 0.05, ^∗∗^*p* < 0.01). Representative data from 10 independent experiments for each group.

Flow cytometry analyses showed that LILRB4 was expressed on all the human MDC1, MDC2, and PDC subsets at a high level in normal pregnancy (**Figures 3A,B**). After *T. gondii* infection, the expression level of LILRB4 was significantly down-regulated on MDC2 and PDC subsets compared with the uninfected group. In normal pregnant mice, LILRB4 was expressed both on CD11c^+^CD8a^-^ and CD11c^+^CD8a^+^ uDC subsets. More importantly, LILRB4 expression on the tolerogenic CD11c^+^CD8a^-^ uDC subset was much higher than that on the CD11c^+^CD8a^+^ uDC subset (**Figures 3C,D**). After *T. gondii* infection, LILRB4 level decreased on the CD11c^+^CD8a^-^ uDC subset (**Figure 3E**) while increased on CD11c^+^CD8a^+^ uDC subset (**Figure 3F**).

### Expression of Functional Molecules on uDCs Was Changed When LILRB4 Is Down-Regulated by *T. gondii* Infection

**FIGURE 4:**
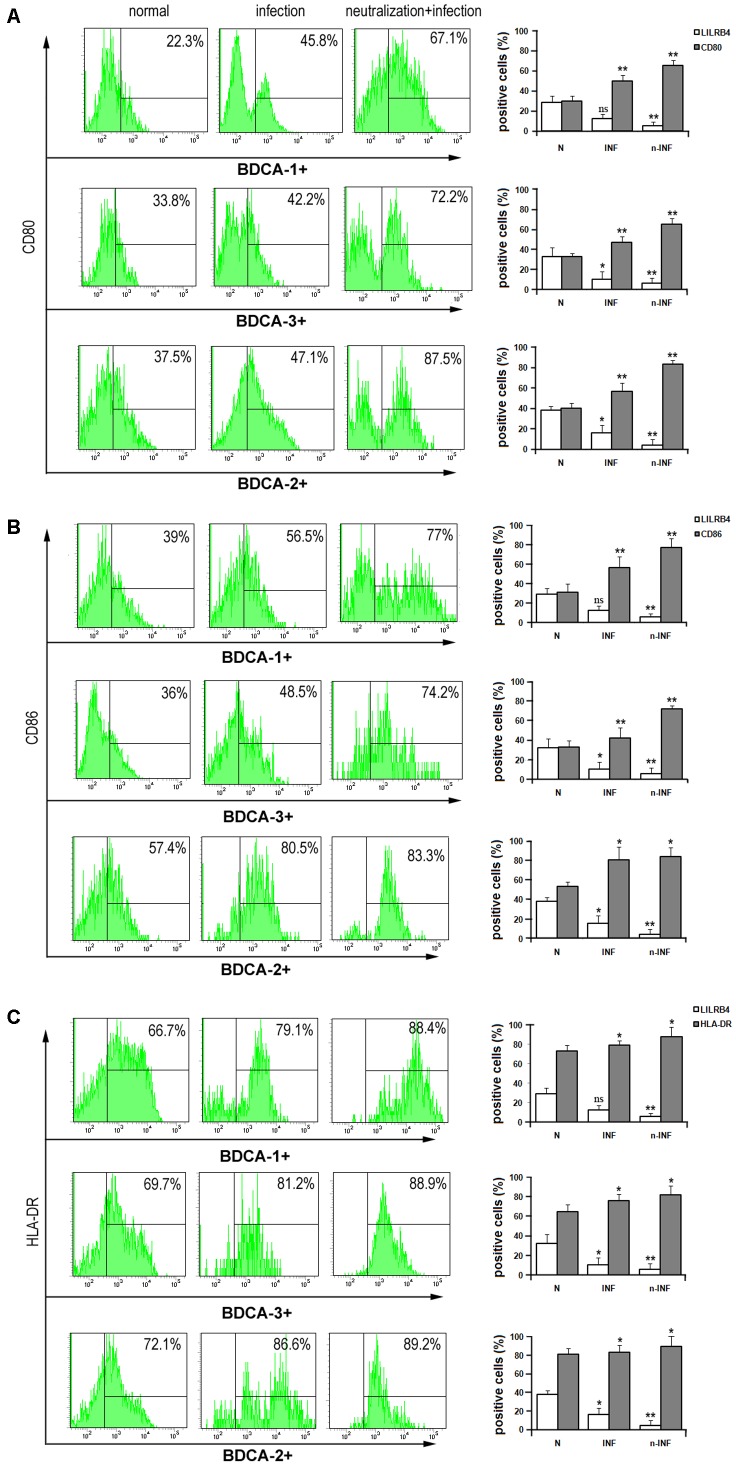
Down-regulation of LILRB4 on human uDC subsets with *T. gondii* infection resulted in changes in the expression of functional molecules of human uDCs. Flow cytometry and histogram analyses of CD80 **(A)**, CD86 **(B)**, HLA-DR **(C)** and LILRB4 expression in BDCA-1^+^, BDCA-2^+^, and BDCA-3^+^ uDC subsets were performed for normal, infected, and anti-LILRB4 neutralizing antibody-treated infected human uDCs. Data are shown as means ± SD (^∗^*p* < 0.05, ^∗∗^*p* < 0.01). Representative data from six independent experiments for each group.

**FIGURE 5:**
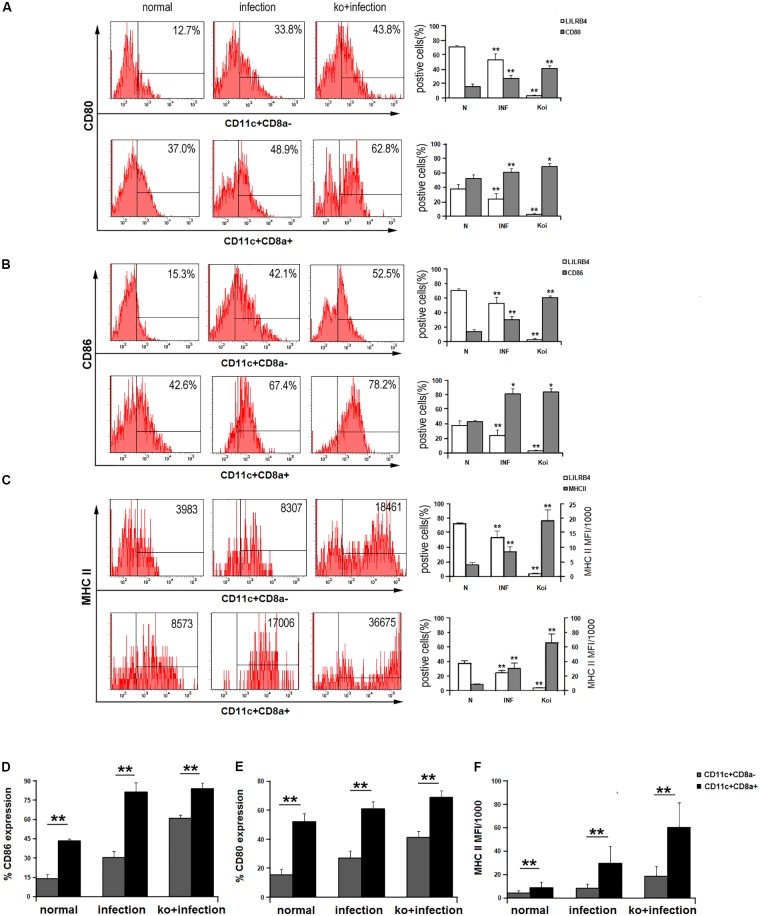
Down-regulation of LILRB4 on mice uDC subsets with *T. gondii* infection resulted in changes in the expression of functional molecules of mice uDCs. CD80 **(A)**, CD86 **(B)**, and MHC II **(C)** followed LILRB4 expression on CD11c^+^CD8a^+^ and CD11c^+^CD8a^-^ uDC subsets was analyzed from normal, infected, and LILRB4^-/-^ infected mice using flow cytometry and histograms. Changes in the expression of CD80 **(D)**, CD86 **(E)**, and MHC II **(F)** followed LILRB4 on CD11c^+^CD8a^+^ and CD11c^+^CD8a^-^ uDC subsets were compared in normal, infected, and LILRB4^-/-^ infected mice. Data are expressed as means ± SD (^∗^*p* < 0.05, ^∗∗^*p* < 0.01). Representative data from 10 independent experiments for each group.

The expression levels of functional molecules CD80, CD86, and HLA-DR on human uDCs subsets were detected (**Figure 4**). After *T. gondii* infection, the expression of CD80, CD86, and HLA-DR were all up-regulated in the three human subsets compared with the uninfected group. On uDCs which LILRB4 was neutralized, CD80, CD86, and HLA-DR were further up-regulated compared with the infected cells. The *in vivo* studies showed that the levels of CD80, CD86, and MHC II on tolerogenic CD11c^+^CD8α^-^ uDC subset were significantly lower than on CD11c^+^CD8α^+^ uDC subset during normal mice pregnancy (**Figure 5**). After *T. gondii* infection, CD80, CD86, and MHC II on murine CD11c^+^CD8α^-^ and CD11c^+^CD8α^+^ uDC subsets were both up-regulated, and they were further increased in LILRB4^-/-^ infected mice compared with the infected mice (**Figures 5A–C**). And the up-regulation of these functional molecules was more obvious on the tolerogenic CD11c^+^CD8α^-^ uDC subset than CD11c^+^CD8α^+^ uDC subset (**Figures 5D–F**).

## Discussion

It was reported that the tolerogenic functions of DCs are characterized by high expression of the inhibitory receptor LILRB4 and low co-stimulatory potential (Chang et al., 2002). During normal pregnancy, LILRB4 can modulate the functions of APCs and plays important roles in immune regulation and tolerance at the maternal–fetal interface (Matsumoto et al., 2001). Although previous studies have shown that LILRB4 is expressed on uDCs during normal pregnancy, the dynamic expression levels remain unclear. In the present study, the level of LILRB4 expression throughout murine gestation was measured by flow cytometry. The results showed that LILRB4 expression was at the lower level in non-pregnant mice uDCs, gradually increased from the first trimester to the middle-late trimester, and peaking on day 14 of gestation in mice. More importantly, LILRB4 expression levels on tolerogenic mice CD11c^+^CD8a^-^ uDC subsets were higher than that on other subsets during normal pregnancy. These data suggest that LILRB4 on uDCs, especially tolerogenic uDC subsets, may be beneficial for normal pregnancy.

Our previous study showed that more severely adverse pregnancy outcomes was observed in *T. gondii-*infected LILRB4^-/-^ pregnant mice, that indicated LILRB4 was related to the development of abnormal pregnancy outcomes following *T. gondii* infection and have found that the expression of LILRB4 on decidual macrophage was detected down-regulated (Li et al., 2017). Whether *T. gondii* infection could affect LILRB4 expression on uDCs and whether this effect subsequently contributes to abnormal pregnancy outcomes were all still unclear. In present study, in order to further investigate whether *T. gondii* infections could affect LILRB4 expression on uDCs subsets, LILRB4 expression was monitored on uDCs during *T. gondii* infection both *in vitro* and *in vivo*. The results showed LILRB4 expression to be significantly down-regulated by *T. gondii* infection on both human and mouse uDCs during pregnancy, and LILRB4 down-regulation were mainly on tolerogenic murine CD11c^+^CD8a^-^ uDC subsets rather than on other uDC subsets. Hence, the results suggest that LILRB4 down-regulation on uDCs, especially tolerogenic uDC subsets, may play a role in the development of *T. gondii*-mediated abnormalities.

To further assess a mechanistic basis for these observations, membrane functional molecules CD80, CD86, and HLA-DR (MHC II in mice) on uDCs were examined during *T. gondii* infection. The functions of DCs are characterized partly by dynamic regulation of co-stimulatory molecules (CD80, CD86) and HLA-DR (McLellan et al., 1995; Nijman et al., 1995). The human decidual MDC1 (BDCA-1^+^) subset expresses low levels of CD80, CD86, and HLA-DR and is regarded as an immature decidual MDC subset involved in inducing immune tolerance (Gardner and Moffett, 2003; Mahnke et al., 2003). MDC2 (BDCA-3^+^) is similar to MDC1 with respect to the phenotype. LILRB4^high^ MDC2, which has a reduced allo-stimulatory capacity is considered a feature of tolerogenic DCs (Velten et al., 2004). PDC (BDCA-2^+^), which has a lower level of CD80 and CD86, was reported to contribute to the maintenance of normal pregnancy at the maternal–fetal interface (Miyazaki et al., 2003). So, the three subsets of u DC all are important for the normal human pregnancy. In the present study, the results showed that CD80, CD86, and HLA-DR in human MDC1, MDC2, and PDC were significantly up-regulated after *T. gondii* infection followed LILRB4 decrease. These data suggested that *T. gondii* infection could significantly weaken the immune tolerogenic function in uDCs by down-regulating LILRB4 expression and enhance immune-activated functions by up-regulating functional molecules CD80, CD86, and HLA-DR expression. To further clarify the role of LILRB4 in the adverse pregnancy outcome caused by *T. gongdii* infection, we performed experiments in which LILRB4 was neutralized with antibody *in vitro to* assess CD80, CD86, and HLA-DR expression. The results showed that functional molecules CD80, CD86, and HLA-DR expression in LILRB4-neutralized infected human MDC1, MDC2, and PDC subsets were further up-regulated compared with the infected uDC subsets. The *in vivo* study showed that, followed the decrease of LILRB4, *T. gondii* infection significantly induced functional molecules CD80, CD86, and MHC II expressions on mice uDCs subsets, and more importantly, the up-regulation of functional molecules were more obvious on tolerogenic CD11c^+^CD8a^-^ uDC subset than CD11c^+^CD8a^+^ uDC subset. The results of *T. gondii-*infected LILRB4^-/-^ pregnant mice that the functional molecules (CD80, CD86, and MHC II) on the two uDC subsets were both further up-regulated compared with the infected WT mice and more obviously on the tolerogenic CD11c^+^CD8a^-^ uDC subset, further confirmed the changes of the functional molecules resulted from LILRB4 down-regulation after *T. gondii* infection. The results demonstrated that the changes in functional molecules (CD80, CD86, and MHC II) are associated with decreased LILRB4 expression on uDCs, especially in tolerogenic uDC subsets, during *T. gondii* infection. A disturbance of uDC tolerance function, due to change in LILRB4 and functional molecules, may contribute to the development of abnormal pregnancy outcomes during *T. gondii* infection.

## Conclusion

The results of this study show that down-regulation of LILRB4 on uDCs, especially on tolerogenic uDC subset, following *T. gondii* infection weakened immune-tolerogenic function of uDC by up-regulating functional molecules CD80, CD86, and HLA-DR (MHC II) expression and ultimately contributed to abnormal pregnancy outcomes by *T. gondii* infection. This investigation further shed light on the molecular immune mechanisms of uDCs in abnormal pregnancy outcomes due to *T. gondii* infection.

## Author Contributions

SZ, JZ, HZ, and XH designed the study and edited the manuscript. SZ, JZ, HZ, and LR performed the mouse experiments. XL, JZ, YJ, CY, LR, and MZ provided the samples and performed the patients experiments. SZ, HZ, and XH wrote the manuscript. SZ, JZ, and HZ made equal contributions to this paper. All authors read the final version of the manuscript and approved it for publication.

## Conflict of Interest Statement

The authors declare that the research was conducted in the absence of any commercial or financial relationships that could be construed as a potential conflict of interest.
